# Faecal carriage of ESBL producing and colistin resistant *Escherichia coli* in avian species over a 2-year period (2017-2019) in Zimbabwe

**DOI:** 10.3389/fcimb.2022.1035145

**Published:** 2022-12-23

**Authors:** Faustinos Tatenda Takawira, Johann D. D. Pitout, Gaetan Thilliez, Tapfumanei Mashe, Ana Victoria Gutierrez, Robert A. Kingsley, Gisele Peirano, Jorge Matheu, Stanley Munyaradzi Midzi, Lusubilo Witson Mwamakamba, David L. Gally, Andrew Tarupiwa, Leckson Mukavhi, Marthie M. Ehlers, Sekesai Mtapuri-Zinyowera, Marleen M. Kock

**Affiliations:** ^1^ Department of Medical Microbiology, University of Pretoria, Pretoria, South Africa; ^2^ National Microbiology Reference Laboratory, Harare, Zimbabwe; ^3^ Department of Microbiology, Alberta Precision Laboratories, Department Pathology and Laboratory Medicine, Cummings School of Medicine, University of Calgary, Calgary, AB, Canada; ^4^ Quadram Institute Biosciences, Norwich, United Kingdom; ^5^ World Health Organization (WHO), Geneva, Switzerland; ^6^ World Health Organization (WHO), Harare, Zimbabwe; ^7^ World Health Organization (WHO), Brazzaville, Republic of Congo; ^8^ Division of Infection and Immunity, The Roslin Institute, The University of Edinburgh, Edinburgh, Scotland, United Kingdom; ^9^ University of Zimbabwe College of Health Sciences, Health Professions Education, Harare, Zimbabwe; ^10^ Tshwane Academic Division, National Health Laboratory Service, Pretoria, South Africa

**Keywords:** *Escherichia coli*, ST155, ST10, ST2197, CTX-M-14, CTX-M-15, ESBL, MCR-1

## Abstract

**Introduction:**

Extended spectrum beta-lactamase (ESBL) producing *Escherichia coli* have become widespread among food producing animals. These strains serve as a reservoir of antibiotic resistance genes (ARGs) and act as a possible source of infection to humans as transmission can occur by direct or indirect contact.

**Methods:**

This study investigated the faecal carriage of ESBL producing and colistin resistant *E. coli* in poultry over a 2-year period (2017-2019) from Zimbabwe. A total of 21 ESBL positive isolates from poultry cloacal specimens were selected for whole genome sequencing from animal *E. coli* isolates bio-banked at the National Microbiology Reference laboratory using phenotypic susceptibility testing results from the National *Escherichia coli* Surveillance Program to provide representation of different geographical regions and year of isolation. Cloacal swabs were collected from 3000 broiler live birds from farm 1 and from farm 2, 40 backyard chickens and 10 ducks were sampled. Antimicrobial susceptibility and ESBL testing were performed as per Clinical Laboratory Standards Institute guidelines. Whole genome sequencing of ESBL producing isolates was used to determine sequence types (STs), ARGs, and phylogroups.

**Results:**

Twenty-one of the included *E. coli* isolates were confirmed as ESBL producers. Three defined sequence type clonal complexes (CCs) were identified (ST10CC, ST155CC and ST23CC), with ST10CC associated with the most antibiotic resistant profile. The ESBL phenotype was linked to the presence of either cefotaximase-Munich-14 (CTX-M-14) or CTX-M-79. Plasmid mediated quinolone resistant determinants identified were *qnrB19* and *qnrS1* and one ST10CC isolate from farm 1 broiler chickens harbored a mobile colistin resistance gene (*mcr-1*). Phylogenetic groups most identified were B1, A and unknown.

**Discussions:**

The avian ESBL producing *E. coli* belonged to a diverse group of strains. The detection of several ARGs highlights the importance of implementing enhanced control measures to limit the spread in animals, environment, and humans. This is the first report of *mcr-1* in Zimbabwe, which further underscores the importance of the One Health approach to control the spread and development of AMR.

## Introduction

Antimicrobial resistance (AMR) surveillance on *Escherichia coli* is a global priority to curb an ever-increasing range of infections caused by bacteria that are no longer susceptible to the common or first –line antibiotics that are used to treat them. The spread of these resistant *E. coli* strains is a serious threat to public health worldwide. The World Health Organization (WHO) has recognised the need for an improved and coordinated global effort to contain AMR (World Health Organization, 2014). The high levels of AMR have been attributed to the extensive and irrational use of antimicrobials in different sectors and has in turn led to the emergence, evolution and spread of numerous antibiotic resistant bacteria (Caruso et al., 2018). The magnitude of antimicrobial usage is expected to increase considerably over the coming years due to the intensification of farming practises in much of the developing world. The AMR in foodborne pathogens is therefore of concern, especially when antibiotics are widely used on commercial farms and backyard farms (Foster-Nyarko et al., 2021b).

Poultry farming is one of the most widespread animal husbandry industries worldwide. Chickens are the most farmed species, with more than 90 billion tonnes of chicken meat produced per year (Food and Agriculture Organisation, 2020). In Zimbabwe, chickens are mostly reared in the backyard and are regarded as a valuable food source. However, the proximity between backyard poultry and humans may facilitate transmission of pathogens such as *E. coli* between these two host species. There are several antimicrobials that are used to raise poultry in several countries (Agunos et al., 2012; Landoni and Albarellos, 2015) including in Zimbabwe, with the aim to prevent and treat diseases, but also to enhance growth and productivity (Page and Gautier, 2012). The same type of antimicrobials is critical and of high importance for human medicine (World Health Organization, 2017). The AMR in avian pathogens may lead to economic losses, derived from the expenditure on ineffective antimicrobials, as well as the burden of untreated poultry diseases (Nhung et al., 2017).

The emergence and spread of cephalosporin and carbapenem resistance, primarily mediated by plasmid-encoded beta-lactamases, is therefore of great concern (Dagher et al., 2018). Colistin is considered a last resort for the treatment of infections caused by carbapenemase-producing members of the *Enterobacterales* (Perreten et al., 2016). The use of polymyxins (colistin and polymyxin B) has been revived in recent years since it emerged as the drug of choice in the face of the emergence of multidrug-resistant (MDR) Gram-negative bacteria (Qiu et al., 2019). Colistin has been widely used in the animal production industry to enhance productivity and control diseases (Van et al., 2020). This led to the emergence of colistin resistance in *E*. *coli.* Several studies have reported the worldwide dissemination of the plasmid-mediated mobilised colistin resistance gene (*mcr-1*) in humans, animals, and the environment, ever since its first detection in November 2015 in China]. To date, nine different plasmid-mediated *mcr* variants have been described, designated as *mcr-1-9*, isolated from humans, animals, and the environment (Hernández et al., 2015; Carattoli et al., 2017; Nishino et al., 2017).

The incidence of AMR *Enterobacterales* in Africa is high. The high incidence especially of ESBL producers has been contributed largely by weak regulations in human and animal health practices, lack of policies and regulation regarding disposal of antimicrobials, despite the limited resources. Most studies on the African continent concentrate on the human clinical and community setting (Storberg, 2014; Sangare et al., 2015), while no data on the molecular epidemiology of *E. coli* in animals have been reported from Zimbabwe. As such the aim of this study was to provide baseline data on the faecal carriage of ESBL producing and colistin resistant *E. coli* in avian species from Zimbabwe.

## Results

### Baseline characteristics of sequenced isolates

A total of 21 ESBL positive cloacal samples were obtained from two major farms from Chitungwiza (2 ducks, 6 backyard chickens) and Harare 13 broiler chickens for analysis. All these ESBL positive carriage isolates displayed MDR phenotypes. The most common MDR phenotype was resistance to ampicillin, cefotaxime, ceftriaxone, ciprofloxacin, nalidixic acid, and tetracycline (AMP-CTX-CRO-CIP-NAL-TET) that was observed in 11 isolates as shown in **Table 1**. The isolates were all susceptible to carbapenems.

**Table 1.1:** Characteristics of the ESBL producing *E. coli* isolates from avian species.

Characteristic	Number of isolates of each sequence type clonal complexes (n)																
	ST10CC					UNKNOWN										ST155	ST23
	10(2)	48(2)	215	761	2461	117	127	937	1140	1196	2107	2197 (3)	2732	5123		155(2)	88
**Location of acquisition**																	
Chicken Cloaca	2	2	1	1	1	1	1	1	1	1	1	3	1	1	2		1
**Antimicrobial**																	
AMP	2	2	1	1	1	1	1	1	1	1	1	3	1	1	2		1
CIP	0	2	1	1	1	1	0	1	1	1	1	3	1	1	2		1
CEFTR	0	0	1	0	1	1	1	1	0	1	1	3	1	0	2		0
TET	2	2	1	1	1	1	1	1	1	1	1	3	1	1	2		1
COT																	
ERT	0	0	0	0	0	0	0	0	0	0	0	0	0	0	0		0
NAL	2	2	1	1	1	1	1	1	1	1	1	3	1	1	2		1
CEFTA	0	0	1	0	0	1	1	1	0	1	1	3	1	0	2		0
CEF	0	0	1	0	1	1	1	1	0	1	1	3	1	1	2		0
**PMQR determinants**																	
aac(6’)-lb-cr	0	0	0	0	1	0	0	0	0	0	0	0	0	1	0		0
qnrS	2	2	1	1	1	1	0	0	1	0	1	0	0	1	1		1
qnrB	2	2	1	1	1	1	0	0	0	1	1	0	1	1	0		1
qnrD	0	0	0	0	0	1	0	0	0	0	0	0	0	0	0		0
**Type of ESBL**																	
CTX-M-14	0	0	0	0	0	1	0	0	0	0	0	3	1	1	0		0
CTX-M-15	0	0	0	0	0	0	0	0	0	0	0	0	0	1	0		0
CTX-M-27	0	0	0	0	0	0	0	0	0	0	0	0	0	0	0		0
Other CTX-M	0	0	CTX-M-79 (1)	0	CTX-M-88(1)	CTX-M-55	0	0	0	CTX-M-79	0	0	0	0	CTX-M-55(1)		0
OXA-1, OXA-9 or OXA-10	0	0	0	0	1	0	0	0	0	0	1	0	0	1	0		0
TEM-1	2	2	TEM-32 (1)	1	1	TEM-214	1	TEM-124	1	TEM-214	1	TEM-216	1	1	TEM-214(2)		1
**Phylogenetic group**																	
A	0	1	0	0	1	0	0	0	0	0	0	1	0	0	0		0
B1	0	0	0	0	0	0	0	1	0	1	0	0	0	0	2		0
B2	0	0	0	0	0	0	1	0	0	0	0	0	0	0	0		0
C	0	0	0	0	0	0	0	0	0	0	0	0	0	0	0		0
D	0	0	0	0	0	0	0	0	1	0	0	0	1	0	0		0
G	0	0	0	0	0	1	0	0	0	0	0	0	0	0	0		0
unknown	2	1	1	1	0	0	0	0	0	0	1	0	0	1	0		1

### Genomic assembly, quality control and phylogenetic tree

**Figure 1:**
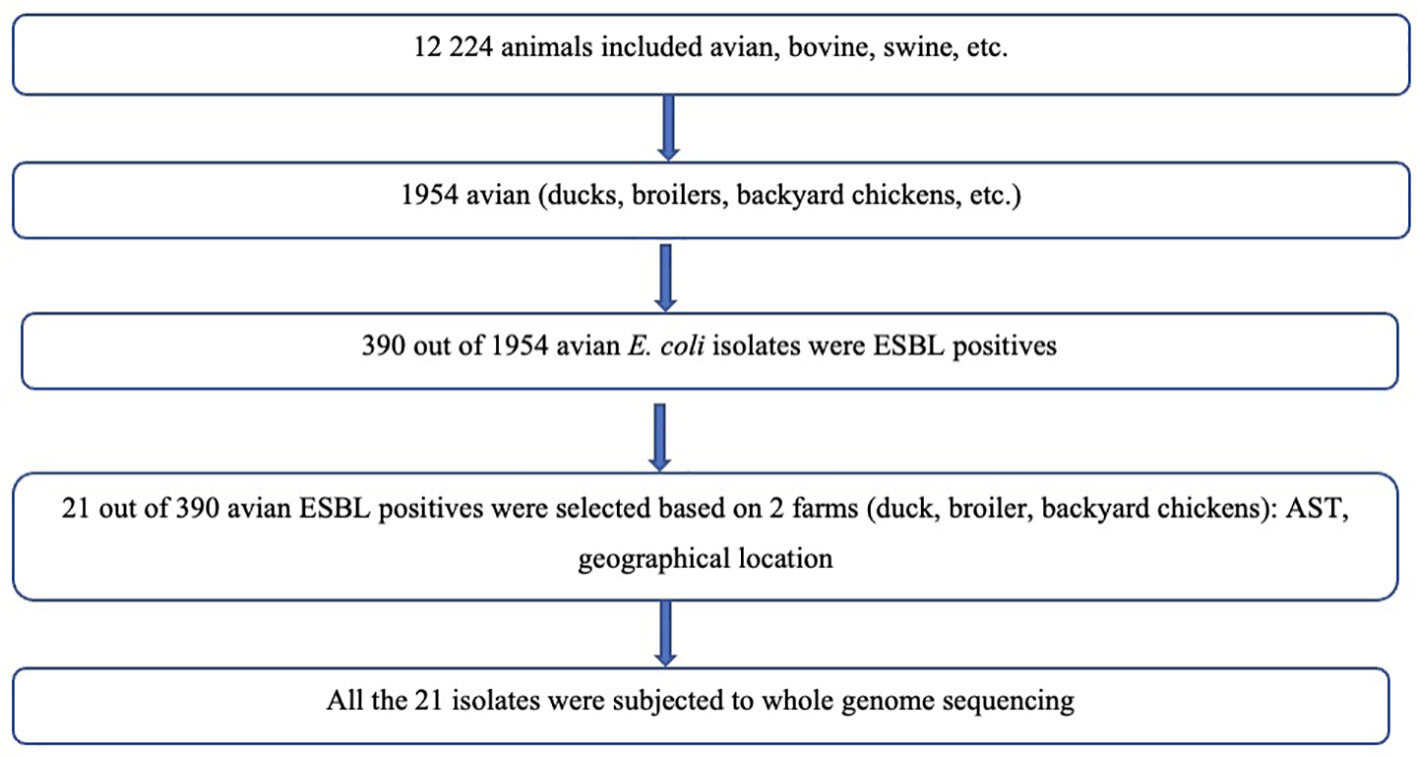
Flow diagram showing selection of bacterial isolates.

**Figure 2:**
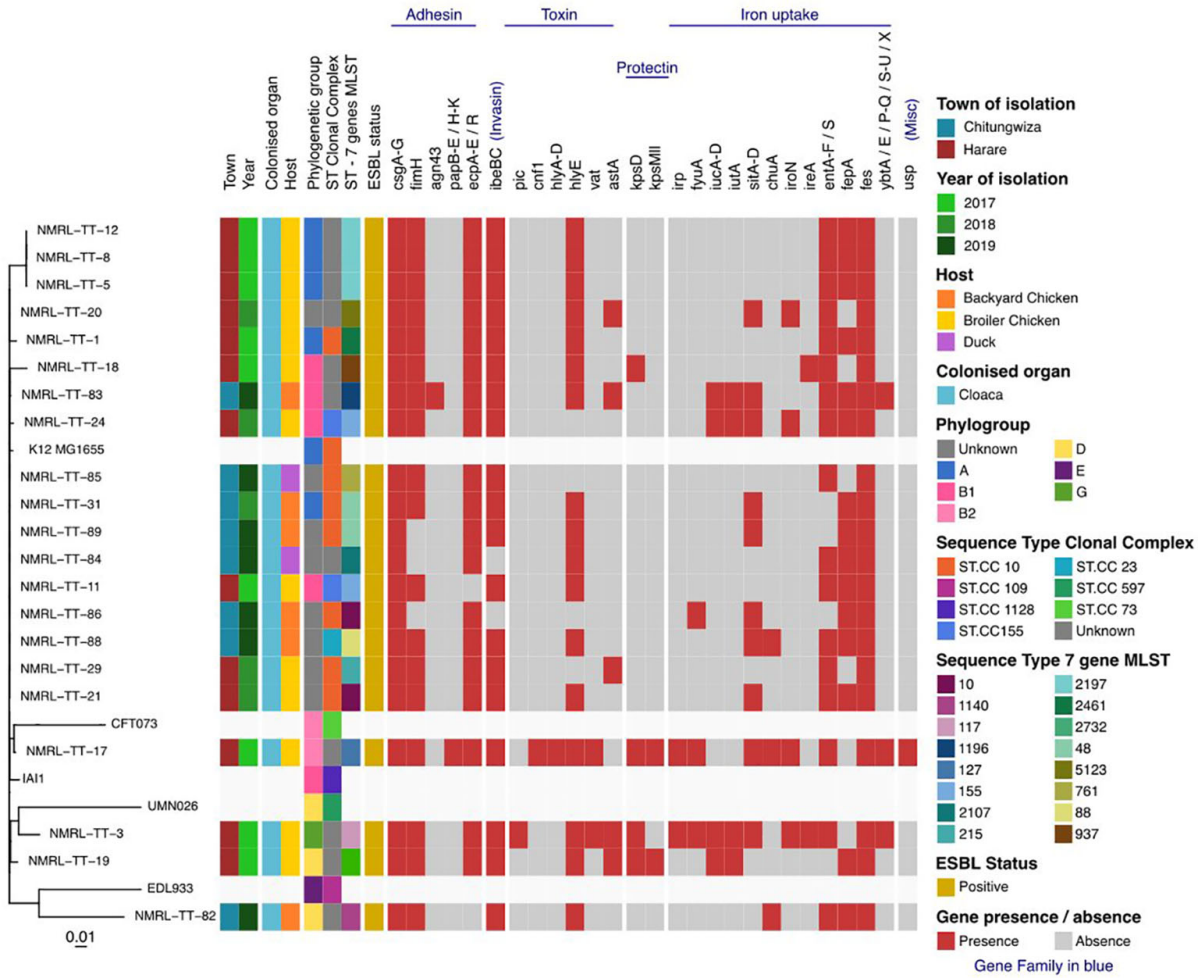
Maximum-likelihood phylogeny of the study isolates reconstructed with RAxML based on non-repetitive, non-recombinant core SNPs.

Twenty-one isolates had a combined length of contigs of assembled genomes ranging from ~4.7 Mbp to 9.8 Mbp, with a minimum contig length required to cover 50% of the genome (N50), ranging between 84 kbp to 340 kbp. The SNP matrix output table with sequenced isolates aligned and compared to reference genomes were used to build a phylogenetic tree as shown in **Figures 1**, **2**.

### Beta-lactamases, plasmid-mediated quinolone resistance determinants and phylogenetic groups

The 21 avian ESBL *E. coli* isolates included in this study presented with diverse ESBL genes, where six harboured the cefotaximase-Munich (CTX-M)-14 gene, one harboured the CTX-M-15 gene, two harboured CTX-M-55, two harboured CTX-M-79 and other Temoneira (TEM) and OXA gene variants. No sulfhydryl variable (SHV) variants were detected as shown in **Table 1**. Illustrates isolates that harboured TEM-1 and OXA-1 genes in addition to CTX-M gene variants. Different plasmid-mediated quinolone resistance determinants were detected in this study, which included the following: *qnrS19* (13), *qnrB*1 (12), *qnrD1* (2) and *aac (6’)-lb-cr* genes respectively. One isolate was positive for the *mcr-*1 gene. Phylogroups identified were as follows: group unknown (8), group A (5); group B1 (4); group D (2); group B2 (1) and group G (1) respectively.

### Multilocus sequence typing

Multilocus sequence typing identified three diverse ST clonal complexes (CCs), which included the following: ST10CC; ST155CC and ST23. Among the ST10CC the STs included ST10, ST48 (2), ST215, ST761, ST2461, while ST155CC included ST155 and ST23CC had ST88. Most of the isolates (12/21, 58%) belonged to one of these defined CCs. The remaining proportion (42%) of isolates in this study represents the undefined clonal complexes referred to as “unknown CCs”. These ST unknown CCs are represented by ST1140, ST1196, ST2197, ST127, ST937, ST5123, ST117, ST2732 and ST2107 (**Table 1**). The farm location, year of collection, antimicrobial susceptibility profile, plasmid mediated quinolone resistant (PMQR) determinants, ESBL types and phylogenetic groups of the above defined and undefined CCs are shown in **Table 1** and **Addendum A - Supplementary File 1 - Table 1.1AA**. All the ESBL isolates were resistant to multiple antimicrobials with most resistant STs being recorded in ST2197, ST10, ST48 and ST155.

### Plasmid replicon types

The most predominant plasmids were Col156, Col440I, ColMG828 and Col pHAD28 that were widely distributed among the ST10 and ST2197 strains. All strains characterised in this study harboured incompatibility group F (IncF) plasmids. The other replicon types detected in this study included IncY, Incl1, Incl2, IncH, IncQ, Inc X, p0111. The plasmid replicons represented as IncFlB were sixteen (n=16) followed by IncFll (n =11) and IncFlA (n = 5).

### Single nucleotide polymorphisms distance comparison of ST10 clonal complex from human isolates to those in avian population

A total of nine isolates [three from human isolates (NMRL-TT-54, NMRL-TT-61 and NMRL-TT-93) and six from poultry isolates (NMRL-TT-1, NMRL-TT-21, NMRL-TT-29, NMRL-TT-31, NMRL-TT-85 and NMRL-TT-86] as shown in **Supplementary File - Table 4** were included for comparison to assess the genetic relatedness of these ST10CC isolates detected in both animals and humans. The snpdist difference between human and poultry isolates ranged between 728 and 17397 SNPs. A comparison of poultry isolates showed SNP differences ranging between 0 and 30 SNPs while human isolates had SNP differences ranging from 908 to 17397 SNPs.

## Discussion

Antimicrobial resistance is one of the biggest threats to food safety and considered a One Health issue with the potential of spreading to other countries, since resistant pathogens do not recognise borders (Mathers et al., 2015). This study reports on the faecal carriage of ESBL producing and colistin resistant *E. coli* in poultry over a 2-year period (2017-2019) in Zimbabwe. The isolates from this study revealed the prevalence of faecal carriage of ESBL *E. coli* in poultry to be 11%, which is comparable to similar studies that were done in León, Nicaragua (13%) (Hasan et al., 2016) and Lebanon (20.6%) (Iman et al., 2018). Extensive use of antimicrobials is recognised as the most important factor selecting for AMR in bacteria (Centers for Disease Control and Prevention et al., 2013) according to the European Surveillance of Veterinary Antimicrobial Consumption (ESVAC) report, sales of veterinary antimicrobial agents in 2016 varied from 0.7 to 2726.5 tons in the 30 participating countries in the European Medicine Agency (EMA) and ESVAC (European Centre for Disease Prevention and Control (ECDC) et al., 2017). Notably, polymyxins were the fifth most sold group of antimicrobials in 2015–2016 (European Centre for Disease Prevention and Control (ECDC) et al., 2017).

A technique called *in silico* MLST was used to determine the circulating clones of importance in the faecal carriage ESBL *E. coli* from Zimbabwean avian species. The most common STs determined were ST10CC, ST155CC and ST88CC, which are known to be interspecies clones (Flament-Simon et al., 2020). Interspecies clones are defined as *E. coli* strains/clones capable of being transmitted among humans, wildlife, companion animals, etc. (Roer et al., 2018). The ST10CC results are consistent with findings from other studies where *E. coli* clones belonging to ST10CC, ST23CC and phylogroup A are increasingly reported in association with ESBL production (Usein et al., 2016; Seni et al., 2021). The ST10CC are known as the largest interspecies clonal complex suggesting that antimicrobial resistant bacteria could be transferred by multiple sources like environment, human or animal contact (Qiu et al., 2019). Given the environment (grass bedding, feeding on kitchen waste and contact with handlers) in which the backyard poultry were exposed to, there is a possibility of transmission of resistant bacteria (Samanta et al., 2018).

Mobile genetic elements (MGEs) play an important role in the transmission of virulence and resistance genes of ESBL producing isolates (Partridge et al., 2018). The different CCs in this study had different ESBL resistance genes ranging from *bla*_TEM_, *bla*_OXA_, and *bla*_CTX-M_ variants and virulence genes. The following virulence genes were detected in this study and includes adhesion factors, evasion/invasion genes, toxins, iron acquisition genes and genes associated with serum resistance. These virulence genes are encoded by *fimH*, *csg*, *agn43*, *kpsD*, *kpsMll*, *ibeBC, fes*, *fepA*, *iucA*, *gspL*, and *hlyE* and can be transmitted through HGT among different Gram-negative bacteria, thus contributing to diseases in humans should they gain access to the appropriate tissue (Hussain et al., 2019).

All the ESBL isolates in this study harboured *IncF* plasmids, which agrees with other epidemiological studies that revealed an association of *bla*_TEM_, *bla*_OXA_, and *bla*_CTX-M_ genes to *IncF* plasmids (Zhao and Hu, 2013; Sukmawinata et al., 2020). Moreover, genes encoding resistance to aminoglycosides, chloramphenicol, quinolones (*qnr)*, tetracyclines (*tet*), and trimethoprim have been observed to co-exist with *bla*_TEM_, *bla*_OXA_, and *bla*_CTX-M_ variant genes in this study. The co-existence of two or more beta-lactamases in the same strain was observed in ST10 and ST155 isolates as reported in other studies (Salah et al., 2019; Obayiuwana and Ibekwe, 2020). There is little doubt that the overuse of cephalosporins and related compounds in poultry farming has been the driving forces in the persistence and dissemination of ESBL producing bacteria with the use of other antimicrobial compounds also exerting the same selective force (Aslam et al., 2018; Hedman et al., 2020). It is worrisome that commensal *E. coli* bacteria, i.e., ST10 phylogroup A from food producing animals may contribute to the dissemination of ESBL resistance and to the transference of resistance genes to other human pathogenic bacteria such as *Salmonella* spp (Ramos et al., 2020).

Epidemiological studies have shown a close genetic relationship between commensal *E. coli*, APEC and human ExPEC (Foster-Nyarko et al., 2021a). A substantial overlap of phylogenetic groups has been observed between the APEC in this study (A, B1 and unknown) and human ExPEC results (B2 and D) observed from a study by Lee et al., 2016 (Lee et al., 2016). This overlap of phylogenetic groups between humans and chickens is worrying given the high consumption of chicken meat around the world and the virulence properties associated with phylogenetic group B2 strains in chickens (Papouskova et al., 2020).

To the best of our knowledge, this is the first report of the finding of plasmid-mediated colistin resistance (*mcr-*1) in Zimbabwe. The *mcr* 1.1 positive *E. coli* isolate belongs to the ST10 clone. Furthermore, our findings suggest that the diversity among the ST10CC in terms of harboring several AMR determinants, is responsible for the high resistance profiles found in this study. The presence of other AMR determinants on the same plasmid as the one harboring *mcr-1* gene may pose a huge public health threat due to the existing risk of spread of resistance genes e.g., *mcr-1* among bacterial strains, to other food producing animals, environment, and humans (Aworh et al., 2020).

A limitation of this study was the small sample size of only 21 avian ESBL *E. coli* isolates with most isolates selected from Harare; however, results from this study contributed to the baseline molecular information for isolates/clones currently and previously linked to resistance in Zimbabwe. Short reads plasmid profile analysis was explored, which does not give a full description of the plasmids as compared to long read sequencing, although this technique provides some indication of the different plasmids within each isolate. Plasmid conjugation experiments could not be performed due to funding and time constraints, therefore future studies should focus on such experiments to get an in-depth understanding of the characteristics of the ESBL -encoding plasmids and the colistin resistance carrying plasmids. As far as we are aware this is the first study to be done in Zimbabwe to provide baseline data on virulence, antimicrobial resistance, and detection of specific lineages of ExPEC circulating in poultry using WGS.

Extended spectrum beta-lactamase producing *E. coli* and *mcr*-1 gene detection for colistin resistance is a public health concern, since these genes are harboured on MGEs, i.e., plasmids, they can easily spread in communities and lead to establishment of *mcr* resistant clones within *E. coli.* It is well-known that MGEs from *E. coli* can spread to other *Enterobacterales* like *Klebsiella pneumoniae.* Since Zimbabwe is a significant poultry importer in Africa, research on this aspect should be widely conducted in Zimbabwe and constantly improved. It is essential to monitor colistin-resistant *E. coli* strains for understanding the prevalence of colistin resistance genes in both human and veterinary medicine, including poultry production.

## Materials and methods

### Selection of bacterial isolates for genomic evaluation

A total of 21 cloacal swabs of ESBL positive avian (broiler chickens, backyard chickens and backyard ducks) *E. coli* was selected from the two farms included in this study. The selection was based on phenotypic susceptibility testing results from 12 240 animal *E coli* isolates bio-banked at the National Microbiology Reference Laboratory. The sampling strategy for broiler chickens in farm 1 with 3000 chickens was as follows. The broiler chickens were caged in 20s therefore a total of 150 compartments. One chicken per compartment was randomly selected for cloacal sampling. Therefore 150 samples underwent culturing and antimicrobial resistance testing

This selection provided representation of different geographical region and years of isolation (2017, 2018, 2019). (**Addendum A - Supplementary File 1 - Table 1.1AA**). The sampling locations/geographical regions included were Chitungwiza (2 ducks, 6 backyard chickens) and Harare (13) due to ease of accessibility and convenience as shown in the Flow diagram (**Figure 1**) below. No information on previous use of antibiotics was collected.

### Study population phenotypic antibiotic resistance profiling

All the selected isolates were sub-cultured on MacConkey or eosin methylene blue (EMB) (Mast Group, Merseyside, UK), incubated at 37°C (Memmert ICH110, Memmert, Germany) for 18 to 24 hours and then stored in 20% glycerol broth at -80°C. Antimicrobial susceptibility testing was determined by the Kirby Bauer disc diffusion method using the Clinical Laboratory Standards Institute (CLSI) guidelines (Clinical and Laboratory Standards Institute, 2018). The antimicrobial drugs tested included ampicillin, trimethoprim-sulfamethoxazole, ciprofloxacin, ceftriaxone, tetracycline, ceftazidime, nalidixic acid, cefepime and ertapenem; results were interpreted as described by the CLSI (Clinical and Laboratory Standards Institute, 2018). The presence of ESBLs was confirmed according to the CLSI criteria for ESBL screening and confirmation (Clinical and Laboratory Standards Institute, 2018). *Escherichia coli* ATCC 25922 was used as quality control strain. Additional data included on collection of each isolate were the year, location of isolation, and city (**Addendum A - Supplementary File 1 - Table 1.1AA** **).**

### Genomic DNA isolation and whole genome sequencing

Genomic DNA (gDNA) of avian *E. coli* was extracted using the Wizard^®^ Genomic DNA Purification Kit (Promega, Madison, WI, USA) according to the manufacturer’s instructions (Promega Corporation, 2019) and stored at -20°C. Library preparation was performed using the Nextera XT DNA Library Preparation Kit (Illumina, San Diego, CA, USA) and sequenced on a MiSeq benchtop sequencer (Illumina, San Diego, CA, USA) at Quadram Biosciences Institute, United Kingdom. The data was uploaded to BaseSpace (http://www.basespace.illumina.com) and then converted to FASTQ files.

### Genomic sequence analyses

The sequences were analysed on the Cloud Infrastructure for Microbial Bioinformatics (O’Connor and Moon, 2016). Paired-end short read sequences were concatenated, and quality-checked using FastQC v0.11.7. *De novo* assembly was performed with SPAdes 3.11 (Bankevich et al., 2012), and quality assessed using QUAST 4.5 (Gurevich et al., 2013). Snippy v4.3.2 (Seemann, 2020) was used to generate a core single nucleotide polymorphism (SNP) alignment using default parameters. The complete genome sequence of *E. coli* strain K12 sub-strain MG1655 was used as reference genome (NCBI accession: NC_000913.3). After the core-genome alignment, a reconstruction of maximum likelihood phylogeny with 1000 bootstrap replicates with RAxML v8.2.4 based on a general time-reversible nucleotide substitution model was performed (Stamatakis, 2014; Kozlov et al., 2019). The phylogenetic tree was rooted using *E. fergusonii* as an outgroup (NCBI accession: GCA_000026225.1). The phylogenetic tree was visualised in Figtree v1.4.4 (Beghain et al., 2018) and annotated in RStudio v3.5.2 and Adobe illustrator v 23.0.3 (Adobe Inc. San Jose, California). Recombination was detected and masked using Gubbins before the phylogenetic tree reconstruction (Croucher and Didelot, 2015). The pairwise SNP distances were computed between genomes from the core-genome alignment using snp-dists v0.6 (Seemann, 2018). The ST10 clonal complex isolates identified in a study of 48 ESBL urine isolates from humans (Takawira et al., 2021), were compared to similar (ST10) clonal complex isolates from this study. The assembled draft genomes underwent similar procedures as above to produce pairwise SNP distances (Seemann, 2018; Seemann, 2020).

### Comparative genomics analysis

The assembled draft genomes were used to define the presence of genes and their alleles. The following databases or typing schemes were used to determine: (i) STs (multi-locus sequence typing; Achtman scheme) (Wirth et al., 2006); (ii) phylogenetic groups (ClermonTyper v1.0.0) (Beghain et al., 2018); (iii) resistance genes (ARIBA database) (Hunt et al., 2017); (iv) virulence factors (virulence factor database, VFDB) (Liu et al., 2019); and (v) plasmids (PlasmidFinder) (Wirth et al., 2006).

## Data availability statement

The data presented in this study are deposited in the NCBI BioProject Number PRJNA 799483 with accession numbers listed in the **Supplementary Datasheet 1**.

## Ethics statement

Ethical clearance was obtained from the Faculty of Health Sciences Research Ethics Committee, University of Pretoria (Ethics Reference Number: 782/2018) and the Medical Research Council of Zimbabwe (Approval Number: MRCZ/A/2394).

## Author contributions

All authors provided critical input and contributed to the manuscript writing and approved its final version. Conception or design of the work FT, JP, MK, SZ, TM, LM. Methodology FT, JP, MK, JM, SM, LWM, AT. Formal analysis and interpretation FT, JP, MK, RK, GT, AG, GP, DG, TM. Writing – original draft preparation FT, JP, MK. Writing – review and editing of the article FT, JP, MK, ME, AT, TM. Final approval of the version to be published FT, JP, MK, SZ, TM, LWM, GT, AG, RK, GP, JM, SM, LM, DG, AT, ME. All authors contributed to the article and approved the submitted version.
