# Supplementary material for: Faecal carriage of ESBL producing and colistin resistant Escherichia coli in avian species over a 2-year period (2017-2019) in Zimbabwe
Source: Front Cell Infect Microbiol. 2022 Dec 23;12:1035145. doi: 10.3389/fcimb.2022.1035145 (PMC9816332; doi:10.3389/fcimb.2022.1035145)
Supplement: Supplementary file 2 [file Table_1.docx]

**Addendum A**

**Supplementary file 1**

**Table 1.1AA**:Epidemiological characteristics of ESBL avian isolates.

| **Sample Number** | **Town** | **YEAR** | **Location of colonization** | **Avian type** | **Phylogenetic group** | **ST CC** | **ST type** | **esbl status** |
| --- | --- | --- | --- | --- | --- | --- | --- | --- |
| NMRL-TT-29 | Harare | 2018 | cloaca | Broiler | unknown | ST10 | 215 | positive |
| NMRL-TT-31 | Chitungwiza | 2018 | cloaca | Backyard | A | ST10 | 48 | positive |
| NMRL-TT-82 | Chitungwiza | 2019 | cloaca | Backyard | D | unknown | 1140 | positive |
| NMRL-TT-83 | Chitungwiza | 2019 | cloaca | Backyard | B1 | unknown | 1196 | positive |
| NMRL-TT-84 | Chitungwiza | 2019 | cloaca | Duck | unknown | unknown | 2107 | positive |
| NMRL-TT-85 | Chitungwiza | 2019 | cloaca | Duck | unknown | ST10 | 761 | positive |
| NMRL-TT-86 | Chitungwiza | 2019 | cloaca | Backyard | unknown | ST10 | 10 | positive |
| NMRL-TT-88 | Chitungwiza | 2019 | cloaca | Backyard | unknown | ST23 | 88 | positive |
| NMRL-TT-89 | Chitungwiza | 2019 | cloaca | Backyard | unknown | ST10 | 48 | positive |
| NMRL-TT-11 | Harare | 2017 | cloaca | Broiler | B1 | ST155 | 155 | positive |
| NMRL-TT-12 | Harare | 2017 | cloaca | Broiler | A | unknown | 2197 | positive |
| NMRL-TT-17 | Harare | 2017 | cloaca | Broiler | B2 | unknown | 127 | positive |
| NMRL-TT-18 | Harare | 2017 | cloaca | Broiler | B1 | unknown | 937 | positive |
| NMRL-TT-19 | Harare | 2017 | cloaca | Broiler | D | unknown | 2732 | positive |
| NMRL-TT-1 | Harare | 2017 | cloaca | Broiler | A | ST10 | 2461 | positive |
| NMRL-TT-20 | Harare | 2018 | cloaca | Broiler | unknown | unknown | 5123 | positive |
| NMRL-TT-21 | Harare | 2018 | cloaca | Broiler | unknown | ST10 | 10 | positive |
| NMRL-TT-24 | Harare | 2018 | cloaca | Broiler | B1 | ST155 | 155 | positive |
| NMRL-TT-3 | Harare | 2017 | cloaca | Broiler | G | unknown | 117 | positive |
| NMRL-TT-5 | Harare | 2017 | cloaca | Broiler | A | unknown | 2197 | positive |
| NMRL-TT-8 | Harare | 2017 | cloaca | Broiler | A | unknown | 2197 | positive |
| UMN026 | N/A | N/A | N/A | N/A | D | ST597 |  | N/A |
| EDL933 | N/A | N/A | N/A | N/A | E | ST109 |  | N/A |
| CFT073 | N/A | N/A | N/A | N/A | B2 | ST73 |  | N/A |
| IAI1 | N/A | N/A | N/A | N/A | B1 | ST1128 |  | N/A |
| Escherichia fergusonii | N/A | N/A | N/A | N/A | fergusonii | ST10300 |  | N/A |
| K12 MG1655 original | N/A | N/A | N/A | N/A | A | ST10 |  | N/A |
