# Supplementary material for: Faecal carriage of ESBL producing and colistin resistant Escherichia coli in avian species over a 2-year period (2017-2019) in Zimbabwe
Source: Front Cell Infect Microbiol. 2022 Dec 23;12:1035145. doi: 10.3389/fcimb.2022.1035145 (PMC9816332; doi:10.3389/fcimb.2022.1035145)
Supplement: Supplementary file 3 [file Table_2.docx]

**Table 1.2AA**:Virulence genes of ESBL avian isolates.

| **Sample Number** | csgA-G | fimH | agn43 | papB-E, H-K | ecpA-E, R | ibeBC | pic | cnf1 | hlyA-D | hlyE | vat | astA | kpsD | kpsMll |
| --- | --- | --- | --- | --- | --- | --- | --- | --- | --- | --- | --- | --- | --- | --- |
| NMRL-TT-29 | yes | yes | no | no | yes | yes | no | no | no | no | no | yes | no | no |
| NMRL-TT-31 | yes | yes | no | no | yes | yes | no | no | no | yes | no | no | no | no |
| NMRL-TT-82 | yes | yes | no | no | no | yes | no | no | no | yes | no | no | no | no |
| NMRL-TT-83 | yes | yes | yes | no | yes | yes | no | no | no | yes | no | yes | no | no |
| NMRL-TT-84 | yes | no | no | no | yes | no | no | no | no | yes | no | no | no | no |
| NMRL-TT-85 | yes | yes | no | no | yes | yes | no | no | no | no | no | no | no | no |
| NMRL-TT-86 | yes | no | no | no | yes | no | no | no | no | no | no | no | no | no |
| NMRL-TT-88 | yes | yes | no | no | yes | yes | no | no | no | yes | no | no | no | no |
| NMRL-TT-89 | yes | no | no | no | yes | yes | no | no | no | yes | no | no | no | no |
| NMRL-TT-11 | yes | yes | no | no | no | yes | no | no | no | yes | no | no | no | no |
| NMRL-TT-12 | yes | yes | no | no | yes | yes | no | no | no | yes | no | no | no | no |
| NMRL-TT-17 | yes | yes | no | yes | yes | yes | no | yes | yes | yes | yes | no | yes | yes |
| NMRL-TT-18 | yes | yes | no | no | yes | yes | no | no | no | yes | no | no | yes | no |
| NMRL-TT-19 | yes | yes | no | no | yes | yes | no | no | no | yes | no | yes | yes | yes |
| NMRL-TT-1 | yes | yes | no | no | yes | yes | no | no | no | yes | no | no | no | no |
| NMRL-TT-20 | yes | yes | no | no | yes | yes | no | no | no | yes | no | yes | no | no |
| NMRL-TT-21 | yes | yes | no | no | yes | yes | no | no | no | yes | no | no | no | no |
| NMRL-TT-24 | yes | yes | no | no | yes | yes | no | no | no | no | no | no | no | no |
| NMRL-TT-3 | yes | yes | no | no | yes | yes | yes | no | no | yes | yes | yes | yes | no |
| NMRL-TT-5 | yes | yes | no | no | yes | yes | no | no | no | yes | no | no | no | no |
| NMRL-TT-8 | yes | yes | no | no | yes | yes | no | no | no | yes | no | no | no | no |
| UMN026 | N/A | N/A | N/A | N/A | N/A | N/A | N/A | N/A | N/A | N/A | N/A | N/A | N/A | N/A |
| EDL933 | N/A | N/A | N/A | N/A | N/A | N/A | N/A | N/A | N/A | N/A | N/A | N/A | N/A | N/A |
|  |  | **adhesins** |  |  |  | invasin |  | **toxins** |  |  |  |  |  | Protectin |

**Table 1.2AA**:Virulence genes of ESBL avian isolates cont..

| **Sample Number** | csgA-G | fimH | agn43 | papB-E, H-K | ecpA-E, R | ibeBC | pic | cnf1 | hlyA-D | hlyE | vat | astA | kpsD | kpsMll |
| --- | --- | --- | --- | --- | --- | --- | --- | --- | --- | --- | --- | --- | --- | --- |
| CFT073 | N/A | N/A | N/A | N/A | N/A | N/A | N/A | N/A | N/A | N/A | N/A | N/A | N/A | N/A |
| IAI1 | N/A | N/A | N/A | N/A | N/A | N/A | N/A | N/A | N/A | N/A | N/A | N/A | N/A | N/A |
| *Escherichi*a *fergusonii* | N/A | N/A | N/A | N/A | N/A | N/A | N/A | N/A | N/A | N/A | N/A | N/A | N/A | N/A |
| K12 MG1655 original | N/A | N/A | N/A | N/A | N/A | N/A | N/A | N/A | N/A | N/A | N/A | N/A | N/A | N/A |
|  |  |  |  |  |  |  |  |  |  |  |  |  |  |  |
| virulence features |  | **adhesins** |  |  |  | invasin |  | **toxins** |  |  |  |  |  | Protectin |

**Table 1.2AA**:Virulence genes of ESBL avian isolates cont..

| **Sample Number** | irp | fyuA | iucA-D | iutA | sitA-D | chuA | iroN | ireA | entA-F, S | fepA | fes | ybtA,E,P-Q, S-U, X | usp |
| --- | --- | --- | --- | --- | --- | --- | --- | --- | --- | --- | --- | --- | --- |
| NMRL-TT-29 | no | no | no | no | no | no | no | no | yes | no | yes | no | no |
| NMRL-TT-31 | no | no | no | no | yes | no | no | no | no | yes | yes | no | no |
| NMRL-TT-82 | no | no | no | no | no | yes | no | no | yes | yes | yes | no | no |
| NMRL-TT-83 | no | no | yes | yes | yes | no | no | no | yes | yes | yes | yes | no |
| NMRL-TT-84 | no | no | no | no | no | no | no | no | yes | yes | yes | no | no |
| NMRL-TT-85 | no | no | no | no | no | no | no | no | yes | no | yes | no | no |
| NMRL-TT-86 | no | yes | no | no | yes | no | no | no | no | yes | yes | no | no |
| NMRL-TT-88 | no | no | no | no | yes | yes | no | no | yes | yes | yes | no | no |
| NMRL-TT-89 | no | no | no | no | yes | no | no | no | no | yes | yes | no | no |
| NMRL-TT-11 | no | no | no | no | no | no | no | no | yes | yes | yes | no | no |
| NMRL-TT-12 | no | no | no | no | no | no | no | no | yes | yes | yes | no | no |
| NMRL-TT-17 | yes | yes | no | no | yes | yes | yes | no | yes | no | yes | yes | yes |
| NMRL-TT-18 | no | no | no | no | no | no | no | yes | yes | no | yes | no | no |
| NMRL-TT-19 | no | no | yes | yes | no | no | no | no | no | yes | yes | no | no |
| NMRL-TT-1 | no | no | no | no | no | no | no | no | yes | yes | yes | no | no |
| NMRL-TT-20 | no | no | no | no | yes | no | yes | no | yes | no | yes | no | no |
| NMRL-TT-21 | no | no | no | no | yes | no | no | no | yes | yes | yes | no | no |
| NMRL-TT-24 | no | no | yes | yes | yes | no | yes | no | yes | yes | yes | no | no |
| NMRL-TT-3 | yes | yes | yes | yes | yes | no | yes | yes | yes | no | yes | yes | no |
| NMRL-TT-5 | no | no | no | no | no | no | no | no | yes | yes | yes | no | no |
| NMRL-TT-8 | no | no | no | no | no | no | no | no | yes | yes | yes | no | no |
| UMN026 | N/A | N/A | N/A | N/A | N/A | N/A | N/A | N/A | N/A | N/A | N/A | N/A | N/A |
| EDL933 | N/A | N/A | N/A | N/A | N/A | N/A | N/A | N/A | N/A | N/A | N/A | N/A | N/A |

| **Sample Number** | irp | fyuA | iucA-D | iutA | sitA-D | chuA | iroN | ireA | entA-F, S | fepA | fes | ybtA,E,P-Q, S-U, X | usp |
| --- | --- | --- | --- | --- | --- | --- | --- | --- | --- | --- | --- | --- | --- |
| NMRL-TT-5 | no | no | no | no | no | no | no | no | yes | yes | yes | no | no |
| NMRL-TT-8 | no | no | no | no | no | no | no | no | yes | yes | yes | no | no |
| UMN026 | N/A | N/A | N/A | N/A | N/A | N/A | N/A | N/A | N/A | N/A | N/A | N/A | N/A |
| EDL933 | N/A | N/A | N/A | N/A | N/A | N/A | N/A | N/A | N/A | N/A | N/A | N/A | N/A |
| UMN026 | N/A | N/A | N/A | N/A | N/A | N/A | N/A | N/A | N/A | N/A | N/A | N/A | N/A |
| EDL933 | N/A | N/A | N/A | N/A | N/A | N/A | N/A | N/A | N/A | N/A | N/A | N/A | N/A |
| CFT073 | N/A | N/A | N/A | N/A | N/A | N/A | N/A | N/A | N/A | N/A | N/A | N/A | N/A |
| IAI1 | N/A | N/A | N/A | N/A | N/A | N/A | N/A | N/A | N/A | N/A | N/A | N/A | N/A |
| *Escherichia fergusonii* | N/A | N/A | N/A | N/A | N/A | N/A | N/A | N/A | N/A | N/A | N/A | N/A | N/A |
| K12 MG1655 original | N/A | N/A | N/A | N/A | N/A | N/A | N/A | N/A | N/A | N/A | N/A | N/A | N/A |
|  |  |  |  |  |  |  |  |  |  |  |  |  |  |
|  |  | iron uptake | |  |  |  |  |  |  |  |  |  | Miscellaneous |

**Table 1.2AA**:Virulence genes of ESBL avian isolates cont..
