# Supplementary material for: Faecal carriage of ESBL producing and colistin resistant Escherichia coli in avian species over a 2-year period (2017-2019) in Zimbabwe
Source: Front Cell Infect Microbiol. 2022 Dec 23;12:1035145. doi: 10.3389/fcimb.2022.1035145 (PMC9816332; doi:10.3389/fcimb.2022.1035145)
Supplement: Supplementary file 4 [file Table_3.docx]

**Addendum A**

**Supplementary file 1**

**Table 1.3AA**:Antimicrobial susceptibility testing results of ESBL avian isolates

| **Sample Number** | **esbl status** | **AMP** | **CIP** | **CEFT** | **TET** | **COT** | **ERT** | **NAL** | **CAZ** |
| --- | --- | --- | --- | --- | --- | --- | --- | --- | --- |
| NMRL-TT-29 | positive | R | R | R | R | R | S | R | R |
| NMRL-TT-31 | positive | R | R | S | R | R | S | R | R |
| NMRL-TT-82 | positive | R | R | R | R | R | S | R | R |
| NMRL-TT-83 | positive | R | R | R | R | R | S | R | R |
| NMRL-TT-84 | positive | R | R | R | R | R | S | R | R |
| NMRL-TT-85 | positive | R | S | R | R | R | S | R | R |
| NMRL-TT-86 | positive | R | R | R | R | R | S | R | R |
| NMRL-TT-88 | positive | R | R | R | R | R | S | R | R |
| NMRL-TT-89 | positive | R | R | R | R | R | S | R | R |
| NMRL-TT-11 | positive | R | R | R | R | R | S | R | R |
| NMRL-TT-12 | positive | R | R | R | R | S | S | R | R |
| NMRL-TT-17 | positive | R | S | R | I | R | S | I | R |
| NMRL-TT-18 | positive | R | R | R | R | S | S | R | R |
| NMRL-TT-19 | positive | R | R | R | R | R | S | R | R |
| NMRL-TT-1 | positive | R | R | R | R | R | S | R | R |
| NMRL-TT-20 | positive | R | R | S | R | R | S | R | R |
| NMRL-TT-21 | positive | R | R | R | R | R | S | S | R |
| NMRL-TT-24 | positive | R | R | R | R | R | S | R | R |
| NMRL-TT-3 | positive | R | R | R | R | R | S | R | R |
| NMRL-TT-5 | positive | R | R | R | R | S | S | R | R |
| NMRL-TT-8 | positive | R | R | R | R | R | S | R | R |
