# Supplementary material for: Faecal carriage of ESBL producing and colistin resistant Escherichia coli in avian species over a 2-year period (2017-2019) in Zimbabwe
Source: Front Cell Infect Microbiol. 2022 Dec 23;12:1035145. doi: 10.3389/fcimb.2022.1035145 (PMC9816332; doi:10.3389/fcimb.2022.1035145)
Supplement: Supplementary file 5 [file Table_4.docx]

**Addendum A**

**Supplementary file 1**

**Table 1.4AA: Single nucleotide polymorphism distances for human ST10 and poultry ST10**

| snp-dists 0.7.0 | Reference | NMRL-TT-1EC | NMRL-TT-21EC | NMRL-TT-29EC | NMRL-TT-31EC | NMRL-TT-54EC | NMRL-TT-61EC | NMRL-TT-85EC | NMRL-TT-86EC | NMRL-TT-93EC |
| --- | --- | --- | --- | --- | --- | --- | --- | --- | --- | --- |
| Reference | 0 | 2195 | 738 | 734 | 749 | 14089 | 5103 | 733 | 728 | 14074 |
| NMRL-TT- 1EC | 2195 | 0 | 1474 | 1476 | 1452 | 14751 | 5550 | 1468 | 1471 | 14718 |
| NMRL-TT-21EC | 738 | 1474 | 0 | 10 | 24 | 13394 | 4386 | 8 | 13 | 13381 |
| NMRL-TT-29EC | 734 | 1476 | 10 | 0 | 30 | 13398 | 4388 | 14 | 17 | 13383 |
| NMRL-TT-31EC | 749 | 1452 | 24 | 30 | 0 | 13402 | 4384 | 16 | 21 | 13391 |
| NMRL-TT-54EC | 14089 | 14751 | 13394 | 13398 | 13402 | 0 | 17370 | 13386 | 13391 | 908 |
| NMRL-TT-61EC | 5103 | 5550 | 4386 | 4388 | 4384 | 17370 | 0 | 4380 | 4385 | 17397 |
| NMRL-TT-85EC | 733 | 1468 | 8 | 14 | 16 | 13386 | 4380 | 0 | 5 | 13375 |
| NMRL-TT-86EC | 728 | 1471 | 13 | 17 | 21 | 13391 | 4385 | 5 | 0 | 13378 |
| NMRL-TT-93EC | 14074 | 14718 | 13381 | 13383 | 13391 | 908 | 17397 | 13375 | 13378 | 0 |
